# Bovine ticks harbour a diverse array of microorganisms in Pakistan

**DOI:** 10.1186/s13071-019-3862-4

**Published:** 2020-01-03

**Authors:** Abdul Ghafar, Alejandro Cabezas-Cruz, Clemence Galon, Dasiel Obregon, Robin B. Gasser, Sara Moutailler, Abdul Jabbar

**Affiliations:** 1 Department of Veterinary Biosciences, Melbourne Veterinary School, The University of Melbourne, Werribee, VIC 3030 Australia; 2 UMR BIPAR, INRAE, ANSES, Ecole Nationale Vétérinaire d’Alfort, Université Paris-Est, 94700 Maisons-Alfort, France; 3 Center for Nuclear Energy in Agriculture, University of Sao Paulo, Piracicaba, 13400-970 Brazil; 4 School of Environmental Sciences, University of Guelph, Guelph, ON N1G 2W1 Canada

**Keywords:** Ticks, Tick-borne pathogens, Co-infections, Cattle, Buffaloes, Microfluidics, Pakistan

## Abstract

**Background:**

Ticks and tick-borne pathogens (TTBP) are a major constraint to livestock production in Pakistan; despite a high prevalence of TTBPs, knowledge on the capacity of Pakistani ticks to carry pathogens and endosymbionts is limited. Furthermore, mixed infections with multiple microorganisms further complicate and limit the detection potential of traditional diagnostic methods. The present study investigated the tick-borne microorganisms in bovine ticks in Pakistan, employing a high-throughput microfluidic real-time PCR based technique.

**Methods:**

Ticks were collected from clinically healthy cattle (*n* = 116) and water buffaloes (*n* = 88) from 30 villages across six districts located in five agro-ecological zones (AEZs) of Pakistan from September to November 2017. The microfluidic real-time PCR was used to test the genomic DNA of individual ticks for the presence of 27 bacterial and eight parasitic microorganisms. Phylogenetic methods were used to assess the genetic relationship of DNA sequences determined herein.

**Results:**

PCR detected DNA of at least one microorganism in each of 221 ticks tested (94.4%, 221/234). DNA-based detection inferred that single pathogens/endosymbionts were the most common (43.4%, 96/221) followed by double (38.9%, 86/221), triple (14.5%, 32/221), quadruple (2.3%, 5/221) and quintuple (0.9%, 2/221) mixed infections. Piroplasms (*Babesia*/*Theileria* spp.) were the most prevalent (31.6%, 74/234), followed by *Ehrlichia* spp. (20%, 47/234) and *Anaplasma marginale* (7.7%, 18/234). *Anaplasma phagocytophilum*, *A. ovis*, *A. centrale*, *Babesia ovis*, *Borrelia* spp., *Rickettsia* spp., *R. massiliae*, *Bartonella* spp. and *Hepatozoon* spp. were also detected. Endosymbionts such as *Francisella*-like (91.5%, 214/234) and *Coxiella*-like (1.3%, 3/234) organisms were also detected in ticks. The highest diversity of microorganisms was detected in *Hyalomma anatolicum* ticks (test-positive for 14/14 microorganisms), followed by *Rhipicephalus microplus* (4/14), *Hy. hussaini* (3/14) and *Rh. annulatus* (2/14). Ticks collected from cattle carried significantly more frequently piroplasms (41.2%, 54/131; *P* < 0.05) than those from buffaloes (19.4%, 20/103). However, the overall prevalence of microorganisms did not vary significantly among ticks from the two host species as well as across different AEZs.

**Conclusions:**

To our knowledge, this is the first study to investigate a wide range of tick-borne microorganisms in bovine ticks using a high-throughput diagnostic method from different AEZs in Pakistan. These findings will aid in establishing the distribution patterns and the control of tick-borne pathogens of bovines in Pakistan.
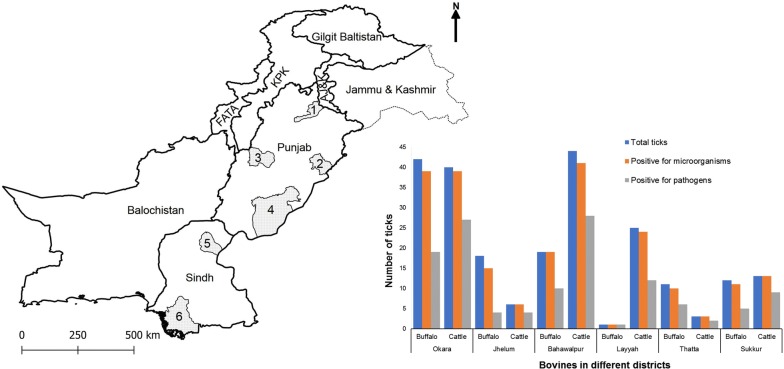

## Background

Ticks (Acari: Ixodida) are obligate, blood-sucking ectoparasites of vertebrates and are distributed worldwide [1]. They pose a major health and production threat to global animal industries [2–4] by affecting their hosts directly by causing irritation, inflammation, anemia, skin/hide damage, toxicosis and paralysis, or indirectly by transmitting a diverse range of pathogens, leading to tick-borne diseases (TBD). It is estimated that 80% of the world’s cattle population, mainly in the tropics and subtropics, is at risk of ticks and tick-borne disease (TTBD) [5]. Furthermore, it is believed that current ongoing climatic and seasonal changes are contributing to the (re)emergence and spread of TTBDs in animals and humans [6].

TTBDs are one of the major health and production constraints for livestock in Pakistan [7] which is the mainstay of Pakistani farmers’ income. Almost 90% of livestock species are kept by small-scale farmers having less than 10 animals, mostly in rural areas [8]. Bovine population is comprised of water buffalo (*Bubalus bubalis*; *n* = 40 million) and cattle (*Bos indicus* and *Bos taurus; n* = 47.8 million) [9]. A recent study on the perceptions of farmers and veterinary health professional on major bovine health, production and welfare constraints in Pakistan revealed that TTBDs are one of the important challenges for cattle and buffalo productivity [Ghafar et al., 2019a, unpublished]. The main bovine ticks reported from Pakistan are *Hyalomma* spp. and *Rhipicephalus* spp. [10–15]. These ticks are responsible for transmitting three important TBDs in cattle and buffaloes, including anaplasmosis (caused by *Anaplasma centrale* and *A. marginale*), babesiosis (caused by *Babesia bigemina* and *B. bovis*) and tropical theileriosis (caused by *Theileria annulata*) [7]. Some of tick-borne pathogens (TBP) transmitted by *Hyalomma anatolicum* are of zoonotic importance (e.g. Crimean Congo haemorrhagic fever) [16].

The occurrence and prevalence of tick-borne pathogens (TBPs) in bovines have been reported from different parts of Pakistan [17–33]. However, most of these studies have utilized conventional diagnostic methods for the detection of TBPs in bovines. Although economical and useful for diagnosing clinical cases, these methods have lower sensitivity and specificity [34]. Furthermore, previous studies were confined to the surrounding areas of major metropolitan cities without considering diverse climatic conditions and bovine production systems in Pakistan which are important factors in the design of epidemiological studies [7]. Recently, several studies have utilized molecular methods for the detection of pathogens in bovines [30, 31] and their ticks [18]. However, these studies only targeted the main TBPs pathogens (e.g. *Anaplasma* spp., *Babesia* spp. and *Theileria* spp.). As ticks usually can carry and transmit multiple pathogens, several non-pathogenic commensals and mutualistic microorganisms, known as endosymbionts, concurrently [35], it is important to explore these non-pathogenic organisms as well because they may interact with pathogens and evolve over time to become pathogenic to humans and/or animals (e.g. *Coxiella burnetii*) [35–37].

In the last decade, various TBPs using molecular methods have been reported from bovines in Pakistan [17, 18, 30, 31]. However, these methods are time-consuming as they can detect only a few pathogens at a time and require large volumes of DNA for the detection of multiple pathogens. Furthermore, very little is known about the occurrence of endosymbionts of ticks from Pakistan which might also play a role in tick species survival and disease ecology in bovines [17]. To address these issues, a novel microfluidic-based high-throughput method has been developed and used for epidemiological and surveillance studies in Europe and South Asia [37–39]. This method uses a small volume of the nucleic acid to perform parallel real-time PCRs on 48.48 or 96.96 chips and can process up to 2304 or 9216 individual reactions, respectively [37, 40].

This study aimed to investigate molecular epidemiology and the prevalence of microorganisms and their co-infections in bovine ticks from five AEZs of Pakistan using a novel microfluidic-based high-throughput method.

## Methods

### Tick collection, identification and DNA extraction

**Fig. 1:**
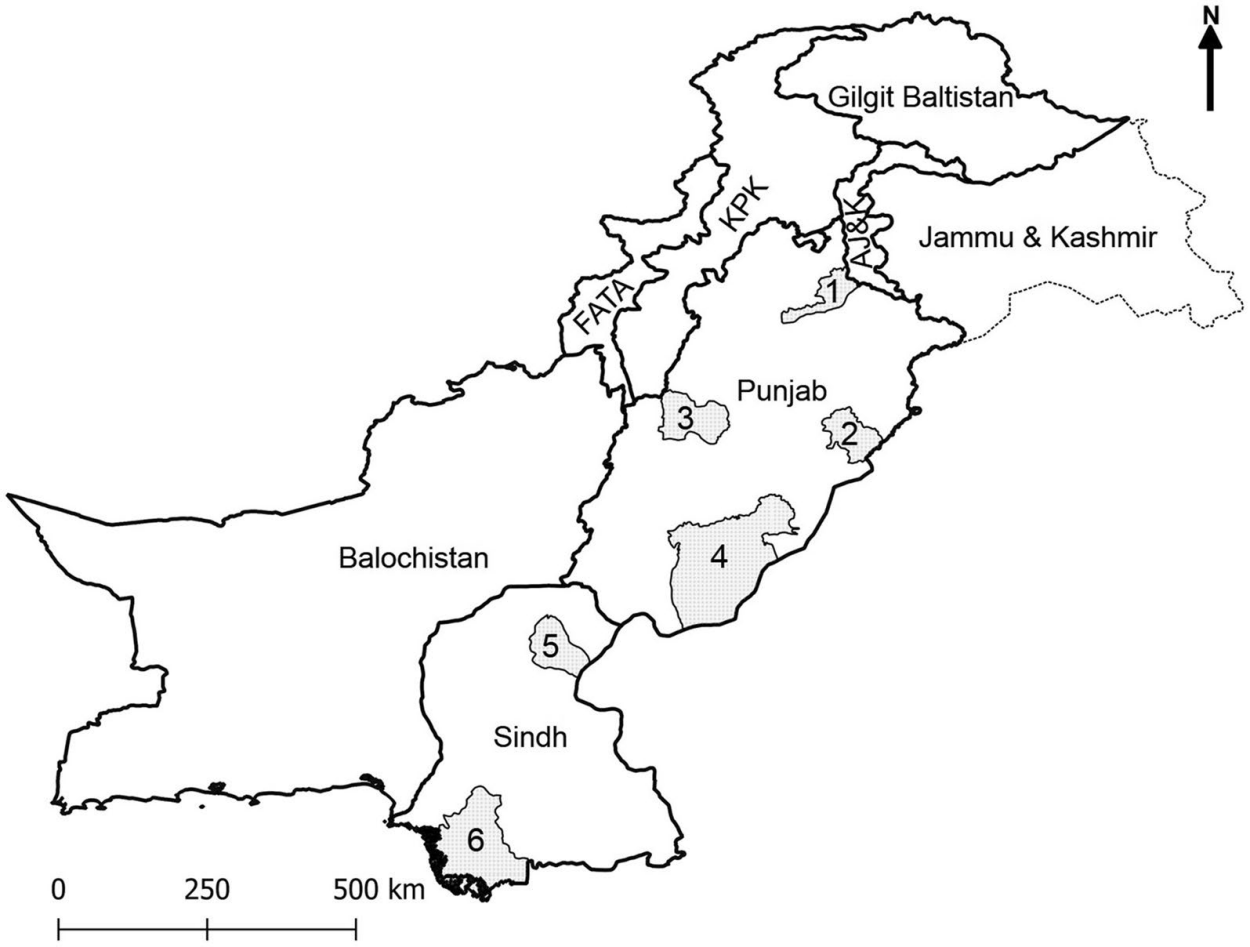
Map of Pakistan showing the districts (grey-coloured areas) included in this study. The names of districts include Jhelum (1), Okara (2), Layyah (3), Bahawalpur (4), Sukkur (5) and Thatta (6). *Abbreviations*: KPK, Khyber Pakhtunkhwa; FATA, Federally Administered Tribal Areas; AJ & K, Azad Jammu and Kashmir

Based on physiography, climate, land use and soil type, Pakistan is divided into 10 AEZs [41]. However, the fodder availability and climatic conditions mainly govern the type of livestock species kept across different AEZs. The bovine population is mainly distributed in two provinces (Punjab and Sindh) of Pakistan. Ticks (*n* = 774) were collected from clinically healthy cattle (*n* = 242) and water buffaloes (*n* = 200) from 30 villages located in six districts of Punjab and Sindh from September to November 2017. These districts are located in five different AEZs and include Bahawalpur (sandy desert), Okara (northern irrigated plain), Jhelum and Layyah (arid; two districts were selected to cover diversity within this zone) districts in Punjab and Sukkur (southern irrigated plain) and Thatta (Indus delta) districts in Sindh (Fig. 1).

Tick specimens from each animal were stored in separate tubes containing 70% ethanol. Subsequently, each tick was morphologically characterized under a dissecting microscope (Olympus SZ40, Japan) using dichotomous keys [42, 43]. Following morphological identification, ticks of the same species from the same animal were pooled in one tube. This resulted in a total of 234 tubes where 131 of those contained ticks from cattle whereas 103 were from buffaloes. DNA was extracted from one tick per tube as per the protocol described previously [Ghafar et al., 2019b, unpublished]. Morphological characterization of ticks was validated using PCR by amplifying cytochrome *c* oxidase subunit 1 (*cox*1) gene, *16S* rRNA gene, and the second internal transcribed spacer and these results have been submitted for publication previously [Ghafar et al., 2019b, unpublished].

### DNA pre-amplification

For DNA amplification, the Perfecta Preamp Supermix (Quanta Biosciences, Beverly, USA) was used according to the manufacturer’s guidelines. Primers (targeted all microorganisms) were pooled combining equal volumes (200 nM final each), and the reaction was performed in a final volume of 5 μl containing 1 μl Perfecta Preamp 5×, 1.25 μl pooled primers mix, 1.5 μl distilled water and 1.25 μl DNA. PCR cycling conditions were one cycle at 95 °C for 2 min followed by 14 cycles at 95 °C for 10 s and 60 °C for 3 min. At the end of the cycling program, the reactions were diluted as 1:10 and the amplicons were stored at – 20 °C until further use.

### Microfluidic real-time PCR

High-throughput microfluidic amplification was performed for major TBPs and potential endosymbionts using 48.48 dynamics array in a Bio-Mark^TM^ real-time PCR system (Fluidigm, California, USA). These chips dispensed 48 samples and 48 PCR mixes into individual wells, followed by on-chip real-time PCR reactions in individual chambers and thermal cycling, resulting in 2,304 individual reactions. For more details regarding the development of this high-throughput tool based on real-time microfluidic PCRs (test of sensitivity, specificity, and controls used), please see Michelet et al. 2014 [37].

Targeted microorganisms (and markers) were *Borrelia* spp. (23S), *Bo. burgdorferi* (*rpoB*), *Bo. garinii* (*rpoB*), *Bo. afzelii* (*fla*), *Bo. valaisiana* (*ospA*), *Bo. lusitaniae* (*rpoB*), *Bo. spielmanii* (*fla*), *Bo. bissettii* (*rpoB*), *Bo. miyamotoi* (*glpQ*), *Bo. mayonii* (*fla*), *Bo. bavariensis* (*pyrG*), *Anaplasma* spp. (*16S*), *A. marginale* (*msp1*), *A. platys* (*groEL*), *A. phagocytophilum* (*msp2*), *A. ovis* (*msp4*), *A. centrale* (*groEL*), *A. bovis* (*groEL*), *Ehrlichia* spp. (*16S*), *E. canis* (*gltA*), *Neorickettsia mikurensis* (*groEL*), *Rickettsia* spp. (*gltA*), *R. conorii* (ITS), *R. slovaca* (ITS), *R. massiliae* (ITS), *R. helvetica* (ITS), *R. aeschlimannii* (ITS), *R. felis* (*orfB*), *Bartonella* spp. (*ssrA*), *Ba. henselae* (*pap31*), *Francisella* spp. (*tul4* and *fopA*), *Coxiella* spp. (*IS1111* and *icd*), *Babesia microti* (*CCTeta*), *B. canis* (*18S*), *B. ovis* (*18S*), *B. bovis* (*CCTeta*), *B. caballi* (*rap1*), *Babesia* str. EU1 (*18S*), *B. divergens* (*hsp70*), *B. vulpes* (*cox*1), *Theileria* spp. (*18S*) and *Hepatozoon* spp. (*18S*). Briefly, amplifications were performed using 6-carboxyfluorescein (FAM)- and black hole quencher (BHQ1)-labelled TaqMan probes with TaqMan Gene expression master mix as per manufacturer’s recommendations (Applied Biosystems, Massachusetts, USA) [37]. PCR cycling conditions comprised of a denaturation step at 95 °C for 5 min followed by 45 cycles at 95 °C for 10 s, 60 °C for 15 s and 40 °C for 10 s. One negative control (water) was included per chip. Detection of ticks’ *16S* gene served as a positive control for the confirmation of DNA extraction. To assess PCR inhibitory molecules present in tick DNA samples, DNA from *Escherichia coli* (EDL933 strain) was added to each sample as an internal inhibition control, and primers and probe specific for the *eae* gene of *E. coli* were used.

### Validation of results by PCR and DNA sequencing

Microfluidic real-time PCR results were confirmed through conventional and nested PCR using different primers (see Additional file 1: Table S1) than those of the BioMark^TM^ system. Amplicons were sequenced by Eurofins MWG Operon (Ebersberg, Germany) and assembled using the Geneious Prime software (Biomatters Ltd, Auckland, New Zealand). An online BLAST (National Center for Biotechnology Information) was used to identify the sequenced organisms.

### Phylogenetic analyses

The partial nucleotide sequences of microorganisms obtained for *18S* rRNA, *16S* rRNA and citrate synthase (*gltA*) genes were used to assess the genetic relationships with those of species of *Babesia*/*Theileria*, *Ehrlichia* and *Rickettsia*, respectively. Reference sequences were downloaded for each pathogen from GenBank and aligned separately (*Babesia*/*Theileria* over 582 bp; *Ehrlichia* over 618 bp; *Rickettsia* over 375 bp) with sequences obtained in this study, using ClustalW [44] in the software MEGA v7.00 [45]. The best-fit evolutionary models for each dataset were selected based on Corrected Akaikeʼs information criterion (cAIC) and Bayesian information criterion (BIC) using MEGA. Phylogenetic trees were constructed using the Neighbour-joining (NJ) and Maximum Likelihood (ML) methods in MEGA and Bayesian Inference (BI) method using Mr Bayes in Geneious Prime [46]. Each Bayesian analysis was run over 20,000,00 generations (ngen = 20,000,00) with two runs and every 400^th^ tree was saved (samplefreq = 400). For the NJ tree estimations, evolutionary distances were computed using the p-distance method whereas for the ML method, initial tree(s) for the heuristic search were obtained automatically by applying Neighbor-Join and BioNJ algorithms to a matrix of pairwise distances estimated using the Maximum Composite Likelihood (MCL) approach, and then selecting the topology with superior log-likelihood value. All positions containing gaps and missing data were eliminated. Bootstrapping method (10,000 replicates) was used to assess the reliability of internal branches and all trees were visualized and edited using MEGA. *Plasmodium falciparum* (GenBank: M19172), *A. marginale* (GenBank: AF414872) and *R. bellii* (GenBank: AY362703) were used as outgroups for *Babesia*/*Theileria*, *Ehrlichia* and *Rickettsia*, respectively.

### Statistical analyses

Chi-square and Fisher’s exact tests were used to evaluate the overall prevalence of microorganisms and to compare their prevalence in different AEZs. Data were analyzed using GraphPad 5 Prism program (GraphPad Software Inc., La Jolla, CA, USA). The multiple correspondence analysis (MCA) was used to analyze the pattern of the association between infections (co-infections) and their distribution across provinces, districts and bovine host species. The inertia values were calculated by standard ‘Burt matrix’ method. The analyses were performed using the software, Statgraphics Centurion v. 16.1.03 (StatPoint technologies Inc, Warrenton, VA).

## Results

### Diversity of microorganisms detected in ticks

**Fig. 2:**
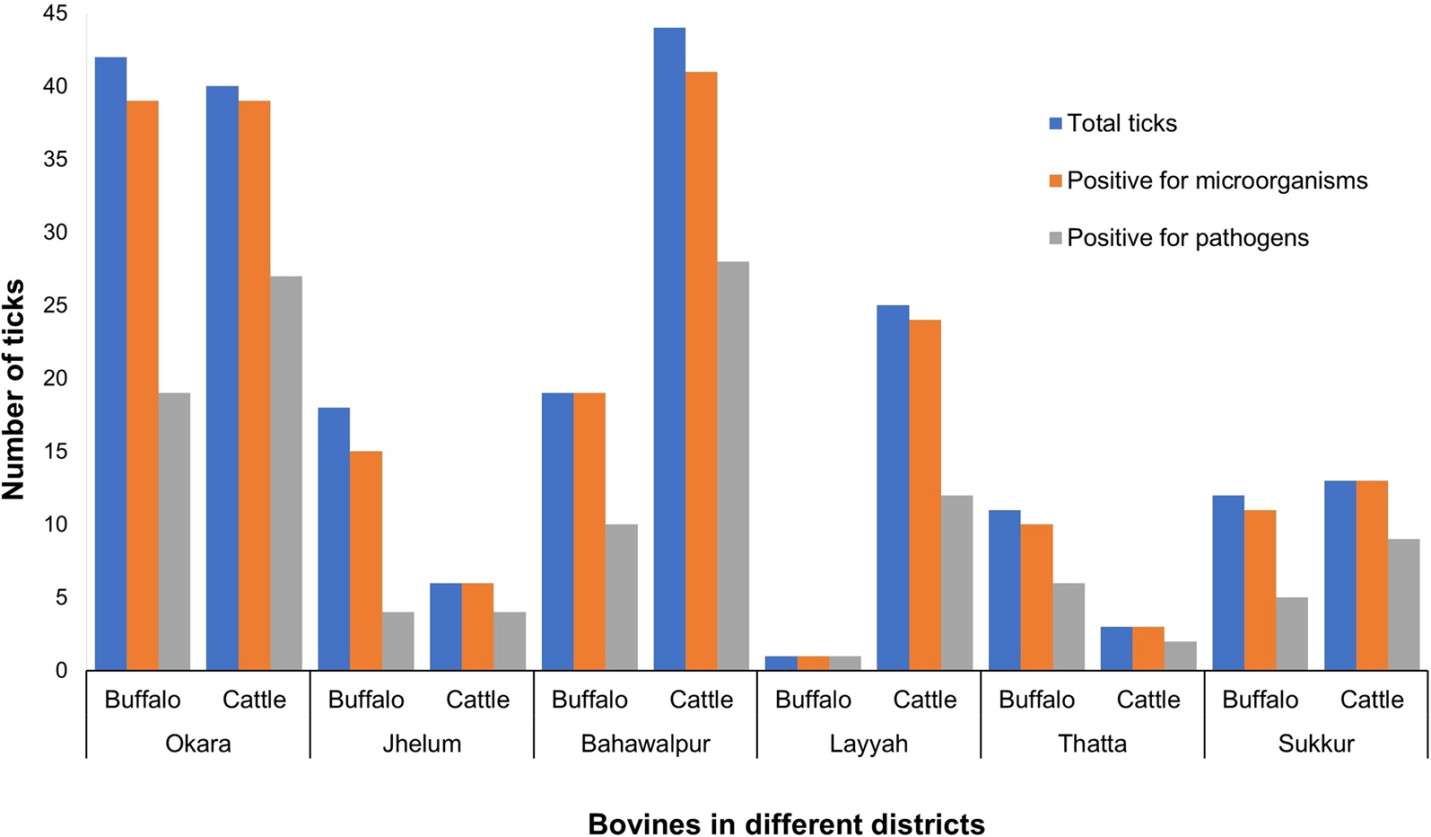
Information about microorganisms detected in ticks collected from bovines in different districts of two provinces, Punjab and Sindh, Pakistan

PCR detected DNA of at least one microorganism in each of 221 ticks tested (94.4%, 221/234) (Fig. 2; Table 1). *Francisella*-like endosymbionts (FLEs) were the most common (91.5%, 214/234) microorganisms detected in ticks (Table 1) followed by piroplasms (31.6%, 74/234), *Ehrlichia* spp. (20%, 47/234), *A*. *marginale* (7.7%, 18/234), *Borrelia* spp. (6.4%, 15/234), *A*. *centrale* (2.6%, 6/234), *Rickettsia* spp. (2.1%, 5/234), *A*. *ovis* (1.7%, 4/234), *R*. *massiliae* (1.7%, 4/234) and *Coxiella*-like endosymbionts (CLEs) (1.3%, 3/234). Furthermore, *A*. *phagocytophilum*, *Bartonella* spp., *Babesia ovis* and *Hepatozoon* spp. were also detected in 0.4% of ticks.

**Table 1:** Diversity of microorganisms in bovine ticks collected from six districts of Pakistan

Microorganism	Punjab								Sindh				Percentage positive (*n*/*N*)
	Okara		Jhelum		Bahawalpur		Layyah		Thatta		Sukkur		
	B (*t* = 42)	C (*t* = 40)	B (*t* = 18)	C (*t* = 6)	B (*t* = 19)	C (*t* = 44)	B (*t* = 1)	C (*t* = 25)	B (*t* = 11)	C (*t* = 3)	B (*t* = 12)	C (*t* = 13)	
*Borrelia* spp.	3	3	2	–	1	4	–	–	–	–	1	1	6.4 (15/234)
*Anaplasma marginale*	4	8	–	2	2	1	–	–	–	–	1	–	7.7 (18/234)
*A. phagocytophilum*	–	–	–	–	–	–	–	–	1	–	–	–	0.4 (1/234)
*A. ovis*	–	–	–	–	1	2	–	–	–	–	–	1	1.7 (4/234)
*A. centrale*	–	3	–	–	1	1	–	1	–	–	–	–	2.6 (6/234)
*Ehrlichia* spp.	12	4	1	–	4	7	1	10	2	1	–	5	20 (47/234)
*Rickettsia massiliae*	–	–	–	–	–	–	–	–	3	1	–	–	1.7 (4/234)
*Rickettsia* spp.	–	–	–	–	–	–	–	–	3	1	1	–	2.1 (5/234)
*Bartonella* spp.	–	1	–	–	–	–	–	–	–	–	–	–	0.4 (1/234)
*Francisella-*like	39	39	14	5	19	40	1	23	7	3	11	13	91.5 (214/234)
*Coxiella-*like	2	1	–	–	–	–	–	–	–	–	–	–	1.3 (3/234)
*Babesia ovis*	–	–	–	–	–	–	–	1	–	–	–	–	0.4 (1/234)
Piroplasms	10	20	1	3	5	21	1	5	1		2	5	31.6 (74/234)
*Hepatozoon* spp.	–	–	–	–	–	–	–	1	–	–	–	–	0.4 (1/234)
Total	70	79	18	10	33	76	3	41	17	6	16	25	

*Abbreviations*: B, Buffalo; C, Cattle; *n*, tested positive ticks; *N*, total tested ticks; *t*, total ticks tested from each bovine host per district

Overall, a high percentage of ticks was test-positive for DNA of microorganisms, with no significant variation (*χ*^2^ = 10.0, *df* = 5, *P* = 0.075) across districts (Sukkur and Layyah, 96%; Okara and Bahawalpur, 95%; Thatta, 93%; Jhelum, 87%) in different AEZs (Table 1). The prevalence of various pathogens (i.e. excluding endosymbionts) in ticks was high across districts (Bahawalpur, 60%; Thatta, 57%; Sukkur, 56%; Okara, 55%; Layyah, 50%; Jhelum, 33%) and it varied significantly (*χ*^2^ = 19.2, *df* = 5, *P* = 0.0018). The highest diversity of microorganisms was found in district Okara where DNA of eight of 14 (57.1%) microorganisms tested for was detected followed by Bahawalpur and Sukkur (7/14) and Layyah and Thatta (6/14) (Table 1). *Ehrlichia* spp., FLEs and piroplasms were found in all districts while *Bartonella* spp. and CLEs were only detected in Okara, *B. ovis* and *Hepatozoon* spp. in Layyah, and *R. massiliae* from Thatta. The prevalence of pathogens in ticks was significantly different between Jhelum and Bahawalpur (*P* = 0.0002), Jhelum and Thatta (*P* = 0.0010), Jhelum and Sukkur (*P* = 0.0017) Jhelum and Layyah (*P* = 0.0214) and Okara and Jhelum (*P* = 0.0027). At the provincial level, the diversity of microorganisms in ticks was higher in Punjab (11/14) than in Sindh (9/14) (Table 1). Based on the bovine host species, cattle ticks carried significantly higher piroplasms (41.2%, 54/131, *P* = 0.0011) than buffalo ticks (19.4%, 20/103); however, the overall prevalence of microorganisms in ticks did not vary significantly between both host species (Table 1).

**Fig. 3:**
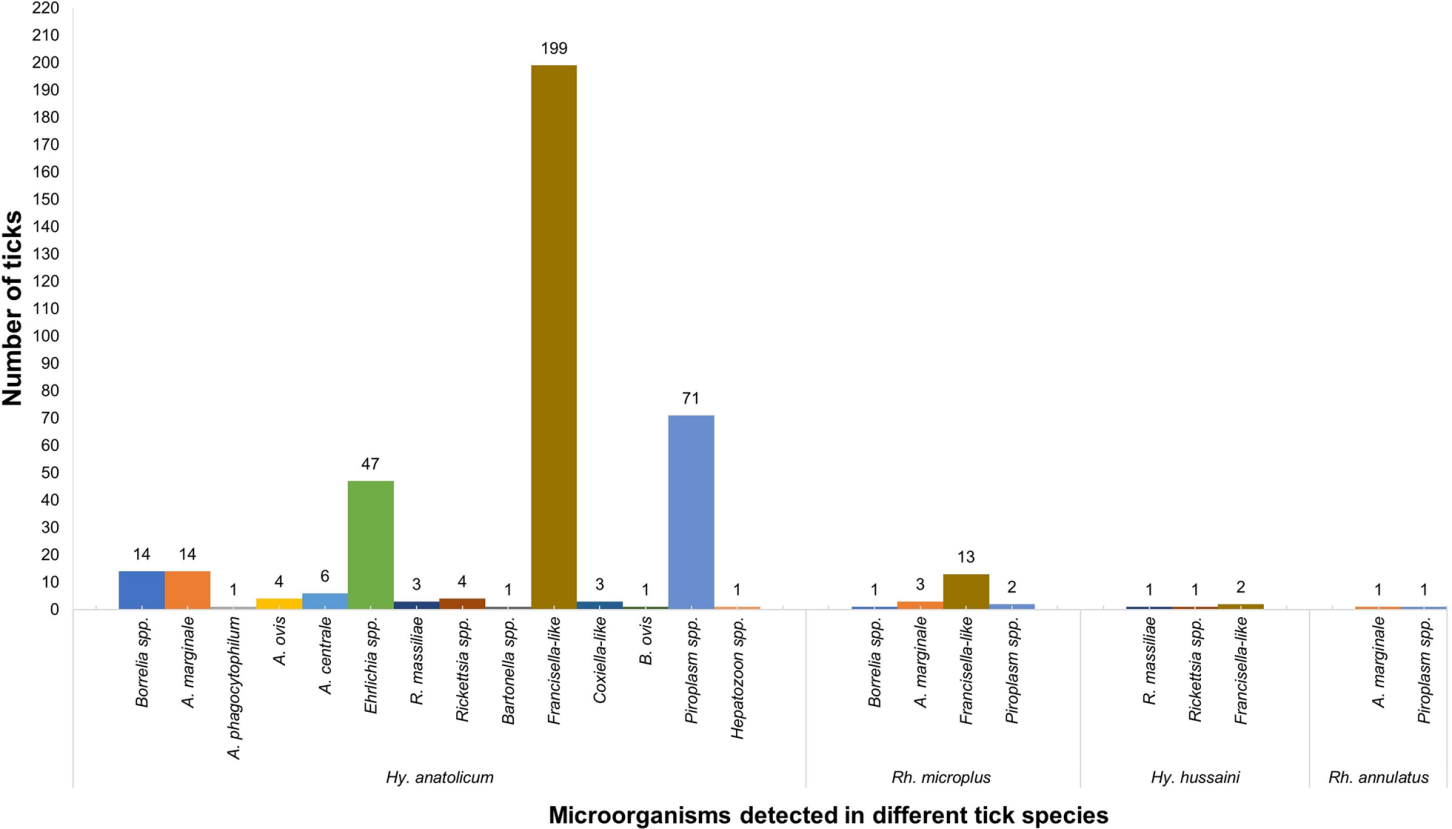
Information about microorganisms detected in different species of ticks collected from bovines in different districts of two provinces, Punjab and Sindh, Pakistan

Among four bovine tick species identified herein, the highest microorganism diversity (14/14) was found in *Hy. anatolicum,* with 96.2% (204/212) of them being test-positive for DNA of at least one microorganism, followed by *Rh. microplus* (4/14), *Hy. hussaini* (3/14) and *Rh. annulatus* (2/14) (Fig. 3, Table 2). However, no microorganism was detected in the single specimen of *Hy. scupense* analyzed (Table 2).

**Table 2:** Diversity of microorganisms in various tick species collected from cattle and buffaloes from the six districts of Pakistan

Tick species	Study district	Bovine host	No. of ticks tested	No. of ticks infected	Detected microorganisms
*Hy. anatolicum*	All six districts^a^	Buffalo	92	88	*Borrelia* spp., *A. marginale*, *A. phagocytophilum*, *A. ovis*, *A. centrale*, *Ehrlichia* spp., *R. massiliae*, *Rickettsia* spp., *Francisella*-like, *Coxiella*-like, piroplasms
		Cattle	120	116	*Borrelia* spp., *A. marginale*, *A. ovis*, *A. centrale*, *Ehrlichia* spp., *Bartonella* spp., *Francisella*-like, *Coxiella*-like, *B. ovis*, piroplasms, *Hepatozoon* spp.
*Hy. hussaini*	Thatta	Buffalo	2	1	*Francisella*-like
		Cattle	1	1	*Francisella*-like, *R. massiliae*, *Rickettsia* spp.
*Hy. scupense*	Jhelum	Buffalo	1	0	–
*Rh. microplus*	Okara and Jhelum	Buffalo	8	6	*Borrelia* spp., *Francisella*-like, piroplasms
		Cattle	9	8	*A. marginale*, *Francisella*-like, piroplasms
*Rh. annulatus*	Jhelum	Cattle	1	1	*A. marginale*, piroplasms
Total			234	221	

^a^Okara, Jhelum, Bahawalpur, Layyah, Thatta, Sukkur

### Co-infections of microorganisms in ticks

Single infections with DNA of various microorganisms were present in 41% (96/234) of ticks, with FLEs (97.9%, 94/96) and piroplasm spp. (2.1%, 2/96) being the main microorganisms detected (Table 3). Among mixed infections of ticks, the highest percentage was found for double infections (36.8%, 86/234) followed by the triple (14.7%, 32/234), quadruple (2.1%, 5/234) and quintuple (0.9%, 2/234) infections. The most common double co-infection was with FLEs and piroplasm species (15.8%, 37/234) followed by FLEs and *Ehrlichia* spp. (9.4%, 22/234) whereas the most common triple co-infection was due to FLEs, piroplasm species and *Ehrlichia* spp. (6.8%, 16/234) (Table 3). *Hyalomma anatolicum* were positive for all types of single as well as mixed infections (i.e. single, double, triple, quadruple and quintuple) while the remaining tick species were positive for up to triple co-infections (Table 2). The distribution of tick infections with one or more microorganisms in various districts varied significantly, and ticks from Okara were positive for all five types of infections while those from Bahawalpur and Layyah had single, double, triple and quadruple infections. Ticks from Jhelum, Sukkur and Thatta were test-positive for DNA of one, two or three microorganisms only (Table 3).

**Table 3:** Occurrence of single and mixed infections of microorganisms in bovine ticks from six districts of Pakistan

Microorganism	Overall prevalence (%)	Proportion of ticks positive for microorganisms					
		Okara (*n* = 82)^a^	Jhelum (*n* = 24)	Bahawalpur (*n* = 63)	Thatta (*n* = 14)	Sukkur (*n* = 25)	Layyah (*n* = 26)
Single infection							
*Francisella*-like	40.2	32	13	22	5	10	12
Piroplasms	0.9	0	1	1	0	0	0
Mixed infection with two microorganisms							
*Francisella*-like + Piroplasms	15.8	12	2	17	0	5	1
*Francisella*-like + *Ehrlichia* spp.	9.4	6	1	5	3	2	5
*Francisella*-like + *Borrelia* spp.	2.1	2	2	0	0	1	0
*Francisella*-like + *Rickettsia* spp.	0.4	0	0	0	0	1	0
*Francisella*-like + *A. marginale*	3.8	6	1	1	0	1	0
*Francisella*-like + *A. centrale*	0.9	1	0	1	0	0	0
*Francisella*-like + *Hepatozoon* spp.	0.4	0	0	0	0	0	1
*Francisella*-like + *Coxiella-*like	0.4	1	0	0	0	0	0
*Ehrlichia* spp. + Piroplasms	0.4	0	0	0	0	0	1
*A. marginale +* Piroplasms	0.4	0	1	0	0	0	0
*Francisella*-like + *A. ovis*	1.3	0	0	2	0	1	0
*Rickettsia* spp. + *R. massiliae*	1.3	0	0	0	3	0	0
Mixed infection with three microorganisms							
*Francisella*-like + Piroplasms + *Borrelia* spp.	1.7	2	0	2	0	0	0
*Francisella*-like + *Ehrlichia* spp. + *Borrelia* spp.	0.9	0	0	1	0	1	0
*Francisella*-like + *Ehrlichia* spp. + *A. centrale*	0.4	0	0	0	0	0	1
*Francisella*-like + Piroplasms + *A. phagocytophilum*	0.4	0	0	0	1	0	0
*Francisella*-like + *Ehrlichia* spp. + Piroplasms	6.8	7	0	4	0	2	3
*Francisella*-like + Piroplasms + *Coxiella-*like	0.4	1	0	0	0	0	0
*Francisella-*like + *Borrelia* spp. + *A. marginale*	0.4	0	0	1	0	0	0
*Francisella-*like + *A. marginale* + Piroplasms	1.3	3	0	0	0	0	0
*Francisella-*like + *A. ovis +* Piroplasms	0.4	0	0	1	0	0	0
*Francisella-*like + *A. centrale* + Piroplasms	0.4	0	0	1	0	0	0
*Francisella*-like + *R. massiliae* + *Rickettsia* spp.	0.4	0	0	0	1	0	0
Mixed infection with four microorganisms							
*Francisella*-like + *Borrelia* spp. + Piroplasms + *Ehrlichia* spp.	0.4	1	0	0	0	0	0
*Francisella-*like + *Borrelia* spp. + *Ehrlichia* spp. + *A. marginale*	0.4	0	0	1	0	0	0
*Francisella-*like + *A. marginale* + *A. centrale* + Piroplasms	0.4	1	0	0	0	0	0
*Francisella-*like + *Ehrlichia* spp. + *Coxiella-*like + Piroplasms	0.4	1	0	0	0	0	0
*Francisella-*like + *Ehrlichia* spp. + *B. ovis* + Piroplasms	0.4	0	0	0	0	0	1
Mixed infection with five microorganisms							
*Francisella-*like + *Borrelia* spp. + Piroplasms + *Ehrlichia* spp. + *A. marginale*	0.4	1	0	0	0	0	0
*Francisella*-like + *A. marginale* + *A. centrale* + *Bartonella* spp. + *Theileria* spp.	0.4	1	0	0	0	0	0
Total positives per district		78	21	60	13	24	25

^a^Number of ticks examined in each district

### Genetic relationship of DNA sequences of selected microorganisms

**Fig. 4:**
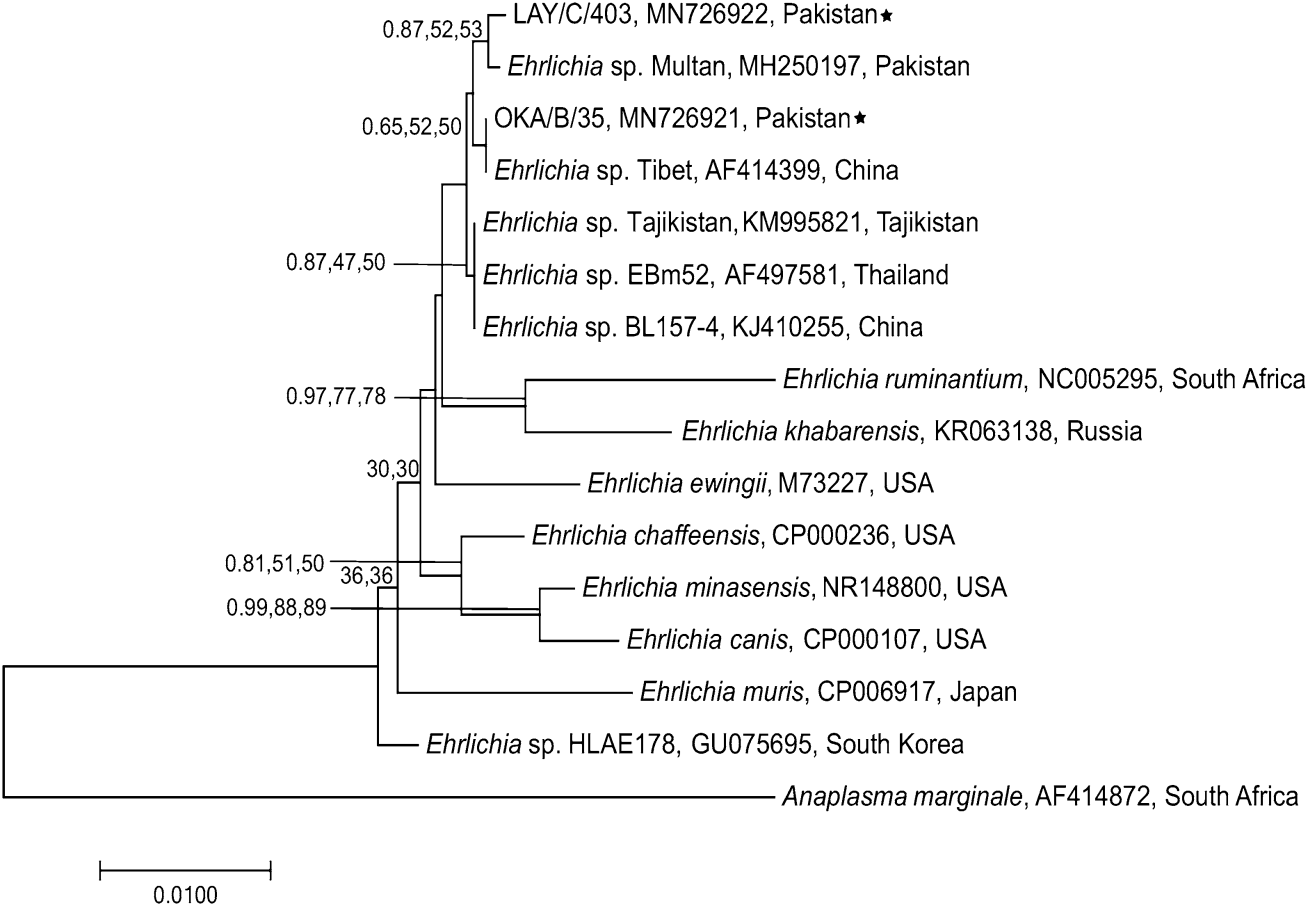
Genetic relationship of *16S* rRNA gene sequences of *Ehrlichia* spp. identified in the present study (starred) with those of *Ehrlichia* spp. available on GenBank. The sequence data (618 bp) were analysed using Neighbour Joining (NJ), Maximum Likelihood (ML) and Bayesian Inference (BI) methods. There was a concordance among the topology of the BI, ML and NJ trees (not shown) and only NJ tree is presented here. Nodal support is given as a posterior probability of BI and bootstrap values for NJ and ML. The tree was rooted using *A. marginale* as outgroup. The scale-bar indicates the number of inferred substitutions per site

**Fig. 5:**
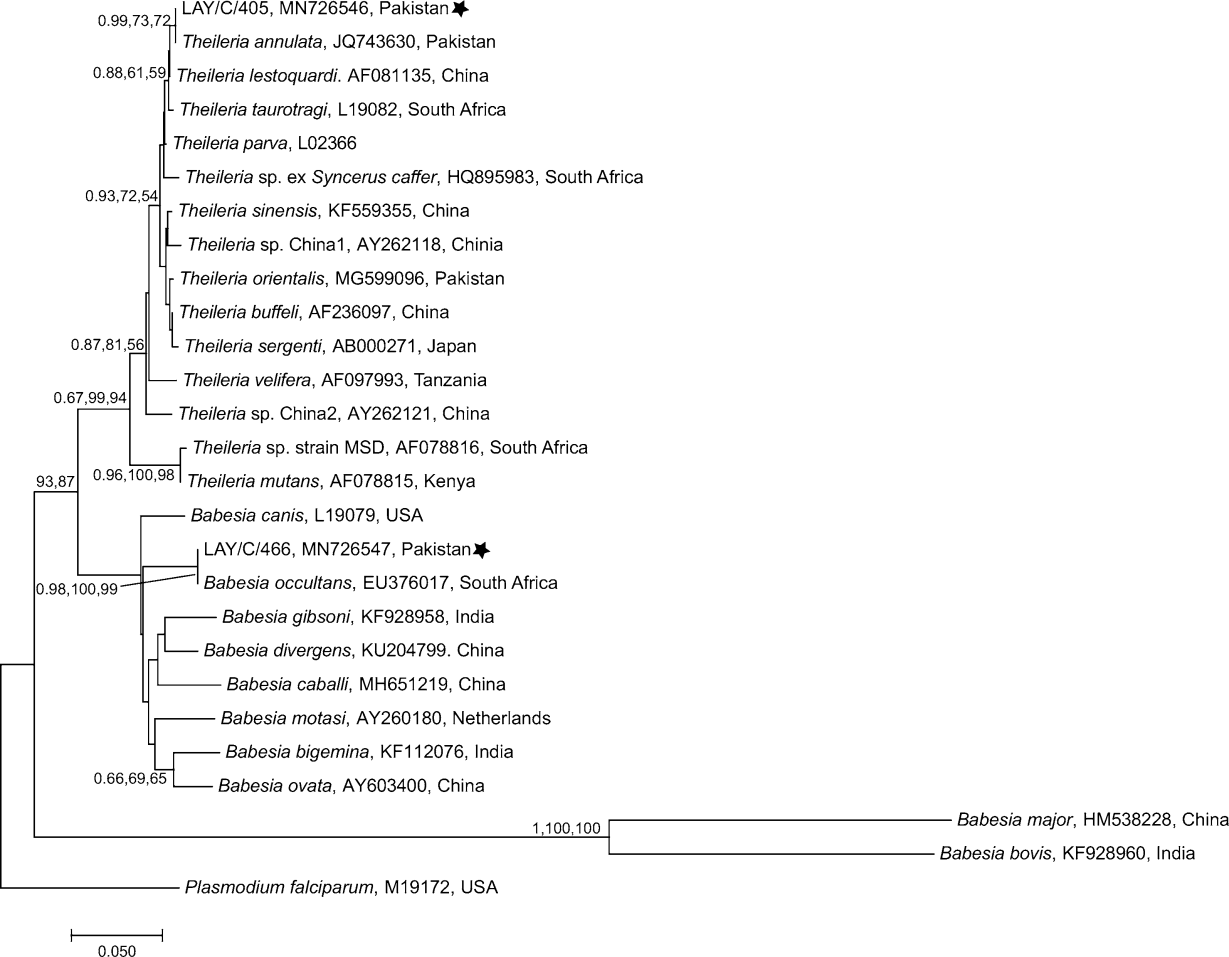
Genetic relationship of *18S* rRNA gene sequences of *Babesia*/*Theileria* spp. identified in the present study (starred) with those of *Babesia*/*Theileria* spp. available on GenBank. The sequence data (582 bp) were analysed using Neighbour Joining (NJ), Maximum Likelihood (ML) and Bayesian Inference (BI) methods. There was a concordance among the topology of the BI, ML and NJ trees (not shown) and only NJ tree is presented here. Nodal support is given as a posterior probability of BI and bootstrap values for NJ and ML. The tree was rooted using *Plasmodium falciparum* as outgroup. The scale-bar indicates the number of inferred substitutions per site

**Fig. 6:**
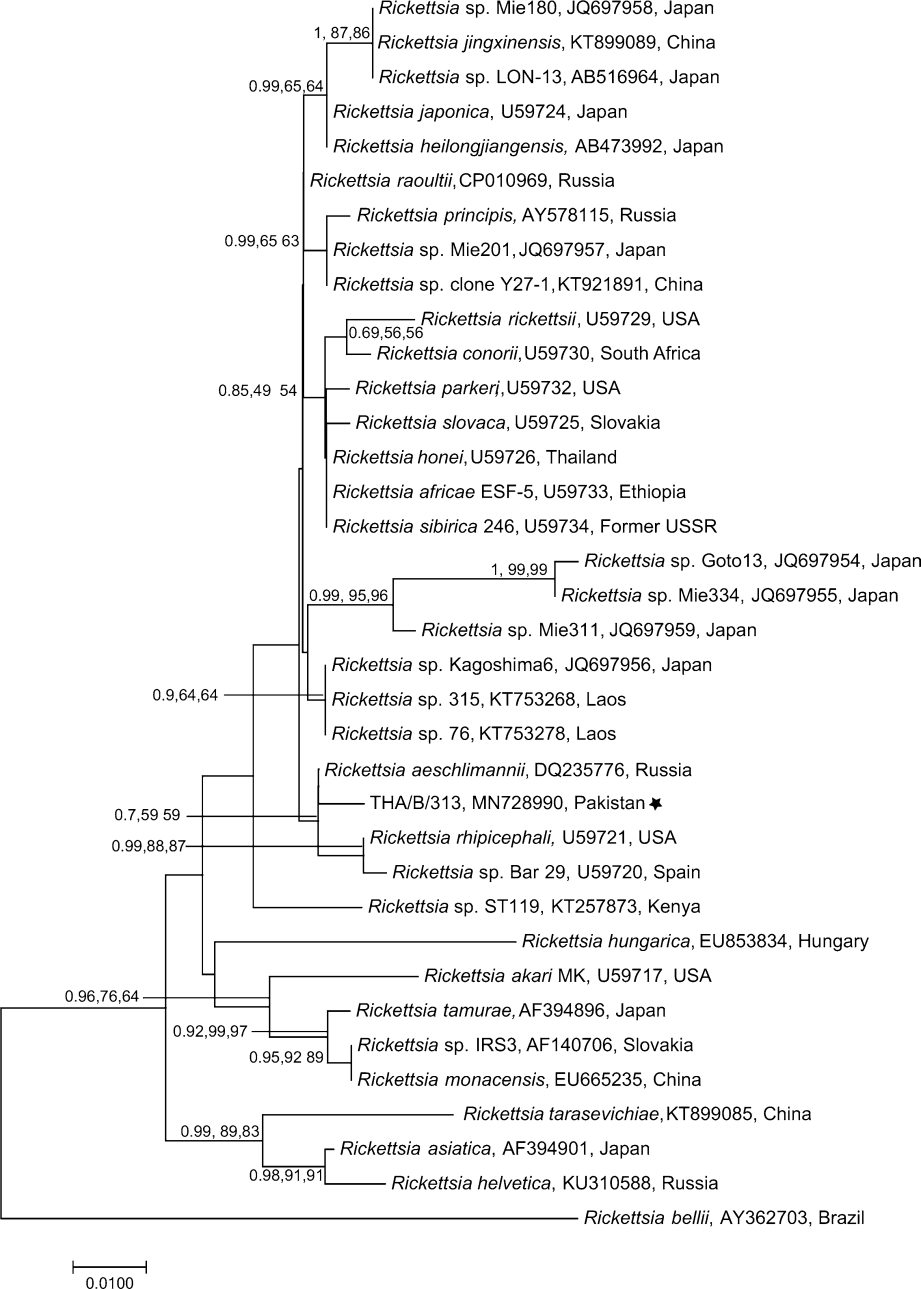
Genetic relationship of *gltA* sequences of *Rickettsia* spp. identified in the present study (starred) with those of *Rickettsia* spp. available on GenBank. The sequence data (375 bp) were analysed using Neighbour Joining (NJ), Maximum Likelihood (ML) and Bayesian Inference (BI) methods. There was a concordance among the topology of the BI, ML and NJ trees (not shown) and only NJ tree is presented here. Nodal support is given as a posterior probability of BI and bootstrap values for NJ and ML. The tree was rooted using *Rickettsia bellii* as outgroup. The scale-bar indicates the number of inferred substitutions per site

Three different phylogenetic methods (BI, ML and NJ) were used to analyze (separately) genetic relationships of *16S* rRNA gene sequences of *Ehrlichia* spp., 18S rRNA gene sequences of *Babesia* and *Theileria* spp. and *gltA* sequences of *Rickettsia* spp. with those of respective previously published sequences. The topologies of all three trees for each target organism were similar; hence, only NJ trees are provided herein (Figs. 4, 5, 6). Two unique *16S* rRNA sequences of *Ehrlichia* spp. (GenBank: MN726921 and MN726922) detected herein grouped with those previously published sequences from Thailand (GenBank:AF497581), Tibet, China (GenBank:AF414399) and Multan, Pakistan (GenBank:MH250197) (Fig. 4), and had a 99.8–100% similarity to these three reference sequences. Two unique *18S* rRNA gene sequences of piroplasms (GenBank: MN726546 and MN726547) were identified and one of these grouped with *T. annulata* sequences from Pakistan (GenBank: JQ743630) and Turkey (GenBank: MK918607), whereas the second sequence clustered with that of *B. occultans* from South Africa (GenBank: U09834) (Fig. 5). For *Rickettsia* spp., only one unique *gltA* sequence (GenBank: MN728990) was found which grouped with *R. aeschlimannii* (GenBank: DQ235776) from Russia, *R. rhipicephali* (GenBank: U59721) from USA and *Rickettsia* sp. Bar (GenBank: U59720) from Spain (Fig. 6).

### Multiple correspondence analyses

Multiple correspondence analysis revealed no correlation between microorganisms and different districts, hosts or AEZs as microorganism clustered close to the centre of multidimensional matrices (Additional file 2: Figure S1, Additional file 3: Figure S2, Additional file 4: Figure S3).

## Discussion

This is the first study to utilize a high-throughput microfluidic technique for the detection of TBPs in bovine ticks collected from five AEZs of Pakistan. This real-time PCR technique offers a unique ability to detect multiple pathogens in ticks [37–39]; here, we detected two protozoan genera (i.e. *Babesia* and *Theileria*) and 12 bacteria (species of *Anaplasma*, *Bartonella*, *Borrelia*, *Coxiella*, *Ehrlichia*, *Francisella*, *Hepatozoon* and *Rickettsia*) of veterinary and/or public health significance. Interestingly, this study revealed that bovine ticks from Pakistan have (i) a diverse range of endosymbionts, with a higher prevalence of FLEs (91.5%); (ii) a high prevalence of TBPs (127 positive of 234 ticks tested); and (iii) a high frequency of co-infections (56.6% of total infections were co-infections). Due to low cycle threshold (Cq) values, genetic characterization was not successful for some of the detected pathogens. As feeding ticks were collected from bovine hosts, we could not justify the bovine or tick origin of detected microorganisms. Likewise, this study does not argue the co-transmission of multiple microorganisms from a tick to the bovine host. However, such studies provide a snapshot of potential TBPs present in a region and help understanding disease(s) dynamics. Furthermore, all ticks tested in this study were collected during one season of the year, and therefore, this study does not inform about the seasonal variation of TBPs.

In this study, we detected the DNA of four *Anaplasma* spp. (*A. centrale*, *A. marginale*, *A. ovis* and *A. phagocytophilum*) in three species of ticks, *Hy. anatolicum*, *Rh. microplus* and *Rh. annulatus*. Bovine anaplasmosis is an endemic TBD in Pakistan, and is mainly caused by *A. marginale* [18, 21, 22]. We found the highest prevalence of *A. marginale* in ticks from Okara district which could be attributed to high prevalence of *Rh. microplus* in this region. Furthermore, this tick is the main vector for *A. marginale* [47]. This study reports the detection of *A. phagocytophilum* DNA in *Hy. anatolicum* for the first time from Pakistan. *Anaplasma phagocytophilum* is a zoonotic TBP infecting several mammalian species and can cause an acute febrile condition in humans, known as human granulocytic anaplasmosis [48]. In Pakistan, *A. phagocytophilum* is mainly responsible for equine granulocytic anaplasmosis and has been reported from equines [49]. Recently, *Anaplasma* species has been reported in bovines from Pakistan [50]. Main tick vectors for the transmission of *A. phagocytophilum* are *Ixodes* spp. but several other tick species have also been found positive for its DNA [51]. Additionally, *A. ovis* was detected in four specimens of *Hy. anatolicum* and it causes ovine anaplasmosis [52–54]. Previously, *A. ovis* has been detected in small ruminants from Khyber Pakhtunkhwa [55] and in three tick species (*Hy. anatolicum*, *Hy. dromedarii* and *Rh. microplus*) from Punjab, Pakistan [18]. The detection of *A. ovis* DNA in multiple tick species indicates the challenges associated with multi-species livestock farming by small-holder dairy farmers.

We found the DNA of *Ehrlichia* species in 47 specimens of *Hy. anatolicum* from all six districts, with the highest percentage in Layyah (11/26) and lowest in Jhelum (1/24). Both districts are located within the same AEZ but hold a diverse topography because Jhelum district has plain and mountainous areas while Layyah district is comprised of desert and plain lands. Furthermore, in Layyah, summer temperatures are higher, and the livestock population there is three times larger than in Jhelum, thereby possibly providing more suitable host and environmental factors for the growth of ticks and the transmission of TBPs. Sequence and phylogenetic analyses of *16S* rRNA gene fragments of *Ehrlichia* spp. detected in this study showed a close similarity to those previously published from China (*Ehrlichia* sp. Tibet; [56]), Pakistan (*Ehrlichia* sp. Multan; [18]) and Thailand (*Ehrlichia* sp. EBm52; [57]) (Fig. 4). Although studies from Pakistan have reported ehrlichiosis in dogs using blood smear examination [58] and molecular tools [38, 59], no information is available on the epidemiology of ehrlichiosis in bovines from Pakistan. Recently, one study investigated TBPs in bovine ticks from Punjab, Pakistan, and reported a high prevalence of *Ehrlichia* spp. (21%) [18]. The high prevalence of *Ehrlichia* spp. in ticks reported in our study as well as in the previous study [18] suggests that *Hyalomma* ticks might be transmitting ehrlichiosis in bovines in Pakistan; however, this hypothesis warrants further investigation.

A high number of piroplasms (*Theileria/Babesia* spp.; 31.6%) were detected in three tick species (i.e. *Hy. anatolicum*, *Rh. microplus* and *Rh. annulatus*). Microfluidic results were validated using piroplasm-specific primers targeting *18S* rRNA gene and revealed that out of seven *Theileria/Babesia-*positive samples sequenced, five identical sequences belonged to *T. annulata* while two remaining identical sequences were identified as *B. occultans*. Since sequencing was not undertaken for all samples, we were not able to determine individual prevalence rates for *Babesia* and *Theileria* spp. in ticks. *Babesia ovis* was also found in one tick sample using species-specific primers. All of these pathogens have been previously reported from Pakistan [18, 30, 31, 60]. Theileriosis is an economically important disease affecting domestic and wild ruminants in tropical and subtropical regions of the world [61]. In Pakistan, this disease is mainly caused by the host cell-transforming sporozoan *T. annulata* and is responsible for high economic losses due to reduced production and mortalities, particularly in exotic and cross-bred animals [7]. Additionally, *T. orientalis* has been reported in bovines and ticks from Pakistan [19, 31] but clinical cases associated with the *Theileria* species complex still remain to be seen. Bovine babesiosis is another important TBD in Pakistan and is mainly caused by *B. bovis* and *B. bigemina* [7]. However, we did not detect *B. bovis* DNA in any tick sample using species-specific primers and no tick was tested for *B. bigemina*, both of which were previously reported to occur in Pakistan [17, 18, 30]. However, during validation using *Babesia*/*Theileria* genera-specific primers, *B. occultans* was found in two *Hy. anatolicum* samples. Since its first detection in South Africa in 1981, *B. occultans* had been considered as apathogenic with its distribution limited to sub-Saharan countries [62, 63]. However, recently it was associated with a clinical babesiosis outbreak in Italy [64] and was also detected in canine blood from India [65] and ticks from Pakistan [18] and China [66]. As climate change can impact the distribution and occurrence of vector-borne diseases [67], *B. occultans* might become an important TBP for bovines in future. Furthermore, we found that ticks from cattle carried significantly higher piroplasms compared to those collected buffaloes which might be due to the natural variation in the susceptibility of the hosts to different pathogens as buffaloes are known to be asymptomatic reservoirs of *Babesia* spp., which are pathogenic to cattle [68].

This study detected, for the first time, DNA of *Borrelia* in 15 tick specimens of *Hy. anatolicum* (*n* = 14) and *Rh. microplus* (*n* = 1) in Pakistan. However, none of the seven species-specific primer pairs employed in this study could verify species identity, and therefore, the pathogenic and/or zoonotic potential of the detected *Borrelia* species could not be established. *Borrelia* species include spirochetes belonging to Lyme borreliosis and relapsing fever spirochete groups as well as intermittent clades and are transmitted by the body louse and hard and soft ticks [69, 70]. There is no surveillance and/or diagnostic system in place for borreliosis in animals or humans in Pakistan; thereby it remained unknown until now. Further testing is required to characterise *Borrelia* spp. and to assess their pathogenic and zoonotic potential.

We found that one *Hy. hussaini* and three *Hy. anatolicum* ticks contained DNA of *R. massiliae*, whereas, five ticks (one *Hy. hussaini* and four *Hy. anatolicum*) were positive for the DNA of unidentified *Rickettsia* spp. DNA sequencing followed by phylogenetic analyses revealed that *gltA* sequences of *Rickettsia* spp. determined herein clustered with those of *R. aeschlimannii* (Fig. 6). This study provides the first report of *Rickettsia* spp. in bovine ticks from Sindh Province of Pakistan as they have previously been detected in ticks from Punjab [18, 71], and Islamabad and Azad Jammu and Kashmir, Pakistan [17]. *Rickettsia massiliae* and *R. aeschlimannii* belong to spotted fever group and both species are of public health significance [72, 73] as reported in the USA, Africa, Asia and Europe [74, 75]. Overall, rickettsial infections rank second after dengue in Southeast Asia as the cause of non-malarial febrile illnesses [76]. However, due to the lack of clinical testing facilities in Pakistan and other developing countries, these infections in animals and humans either remain unreported or underreported. We also found *Bartonella* spp. and *Hepatozoon* spp. in *Hy. anatolicum* ticks from Okara and Layyah districts, respectively. However, species identification of these organisms could not be established, and therefore, the pathogenic or zoonotic potential could not be assessed.

In this study, a high prevalence of endosymbionts such as FLEs (91.5%) was inferred, for the first time, in *Hy. anatolicum*, *Rh. microplus* and *Hy. hussaini* ticks from Pakistan. Previously, a study by Karim et al. [17] investigated tick microbiomes of specimens collected from different livestock species from various parts of Pakistan and reported endosymbionts, *Francisella* (0.2% in *Hy. anatolicum* ticks from buffaloes) and *Coxiella* (7.9% in ticks belonging to the genera *Rhipicephalus*, *Haemphysalis*, *Hyalomma* and *Ornithodoros*) for the first time from Pakistan. Endosymbionts are non-pathogenic mutualistic and/or commensal microbes, and they are also abundant in ticks. Main tick endosymbionts belong to the genera of *Rickettsia*, *Francisella* and *Coxiella* [36, 77]. Endosymbionts can (i) have multiple effects (detrimental or beneficial) on their carriers [77–79]; (ii) cause diseases to humans [80, 81]; and (iii) interact with other TBPs and affect their colonization and transmission [82–84]. The composition of endosymbionts can vary significantly among ticks in different parts of the world and it can be affected by several factors such as environment [85], season [86], geographical location [87], tick species [86], tick life stage [88], feeding status [85] and co-existing pathogens [89]. Given that endosymbionts could play role in the prevalence and transmission of various pathogens, further investigations are required to explore the endosymbiotic communities in ticks infesting animals in Pakistan. Mixed infections with FLEs and piroplasm spp. and/or *Ehrlichia* spp. were most commonly detected in ticks across all AEZs. Ticks co-infected with multiple pathogens might pose a greater risk to animals as well as humans because of increased risk of co-infections which would ultimately increase the clinical complexity of diseases. High co-infection rates of microorganisms in ticks highlights the need of such studies to be conducted on larger populations to further assess pathogen communities and their potential interactions as well as their pathogenic or zoonotic potential.

## Conclusions

This study reports that multiple TBPs of animal and public health significance are carried by bovine tick population in Pakistan. Co-infections with multiple pathogens are common, and endosymbionts are ubiquitously present. Overall, the prevalence of TBPs does not vary significantly across different AEZs of the country, but some pathogens may be restricted to particular regions or AEZs. Future studies are required to characterize endosymbionts further to explore their possible interaction(s) with pathogens transmitted by ticks collected from a larger animal population across different seasons in various AEZs.

## Supplementary information

**Additional file 1: Table S1.** Primers used for validation of microfluidic real-time PCR results.

**Additional file 2: Figure S1.** Multiple correspondence analyses maps from the projections of the first two dimensions, showing the associations between infections (co-occurrence), and their distributions among six districts in Punjab and Sindh provinces of Pakistan. Percentage in each dimension (axis) indicates the fraction of the inertia that each principal component explains. Analyses are shown for correlation between infections and their prevalence in different provinces.

**Additional file 3: Figure S2.** Multiple correspondence analyses maps from the projections of the first two dimensions, showing the associations between infections (co-occurrence), and their distributions among six districts in Punjab and Sindh provinces of Pakistan. Percentage in each dimension (axis) indicates the fraction of the inertia that each principal component explains. Analyses are shown for correlation between infections and their prevalence in different districts.

**Additional file 4: Figure S3.** Multiple correspondence analyses maps from the projections of the first two dimensions, showing the associations between infections (co-occurrence), and their distributions among six districts in Punjab and Sindh provinces of Pakistan. Percentage in each dimension (axis) indicates the fraction of the inertia that each principal component explains. Analyses are shown for correlation between infections and their prevalence in bovine host species.

## Data Availability

All data generated or analyzed during this study are included in this published article.
