# Supplementary material for: Bovine ticks harbour a diverse array of microorganisms in Pakistan
Source: Parasit Vectors. 2020 Jan 3;13:1. doi: 10.1186/s13071-019-3862-4 (PMC6942265; doi:10.1186/s13071-019-3862-4)
Supplement: Supplementary file 1 — Additional file 1: Table S1. Primers used for validation of microfluidic real-time PCR results. [file 13071_2019_3862_MOESM1_ESM.docx]

**Additional file 1: Table S1** Primers used for validation of microfluidic real-time PCR results

| Species | Target gene | Forward primer | Reverse primer | Reference |
| --- | --- | --- | --- | --- |
| *Babesia*/*Theileria* | 18S rRNA | GAGGTAGTGACAAGAAATAACAATA | TCTTCGATCCCCTAACTTTC | [1] |
| *Anaplasma*/ *Ehrlichia*/ | 16S rRNA | GAACGAACGCTGGCGGCAAGC | AGTAYCGRACCAGATAGC CGC | [2] |
| *Rickettsia* spp. | Citrate synthase | GGGGGCCTGCTCACGGCGG | ATTGCAAAAAGTACAGTGAAC | [3] |
| *Hepatozoon* spp. | 18S rRNA | ATACATGAGCAAAATCTCAAC | CTTATTATTCCATGCTGCAG | [4] |
| *Bartonella* spp. | Citrate synthase | GGGGACCAGCTCATGGTGG | AATGCAAAAAGAACAGTAAACA | [5] |

**References**

1. Gubbels J, De Vos A, Van der Weide M, Viseras J, Schouls L, De Vries E, et al. Simultaneous detection of bovine *Theileria* and *Babesia* species by reverse line blot hybridization. J Clin Microbio. 1999;37:1782-9.

2. Rar VA, Fomenko NV, Dobrotvorsky AK, Livanova NN, Rudakova SA, Fedorov EG, et al. Tickborne pathogen detection, western Siberia, Russia. Emerg Infect Dis. 2005;11:1708.

3. Regnery RL, Spruill CL, Plikaytis B. Genotypic identification of *Rickettsiae* and estimation of intraspecies sequence divergence for portions of two rickettsial genes. J Bacteriol. 1991;173:1576-89.

4. de Azevedo Gomes L, Moraes PHG, do Nascimento LdCS, O’Dwyer LH, Nunes MRT, Rossi AdRP, et al. Molecular analysis reveals the diversity of *Hepatozoon* species naturally infecting domestic dogs in a northern region of Brazil. Ticks Tick Borne Dis. 2016;7:1061-6.

5. Norman A, Regnery R, Jameson P, Greene C, Krause D. Differentiation of *Bartonella*-like isolates at the species level by PCR-restriction fragment length polymorphism in the citrate synthase gene. J Clin Microbiol. 1995;33:1797-803.
