# Supplementary figures and images for: Bovine ticks harbour a diverse array of microorganisms in Pakistan
Source: Parasit Vectors. 2020 Jan 3;13:1. doi: 10.1186/s13071-019-3862-4 (PMC6942265; doi:10.1186/s13071-019-3862-4)

## Slide 1
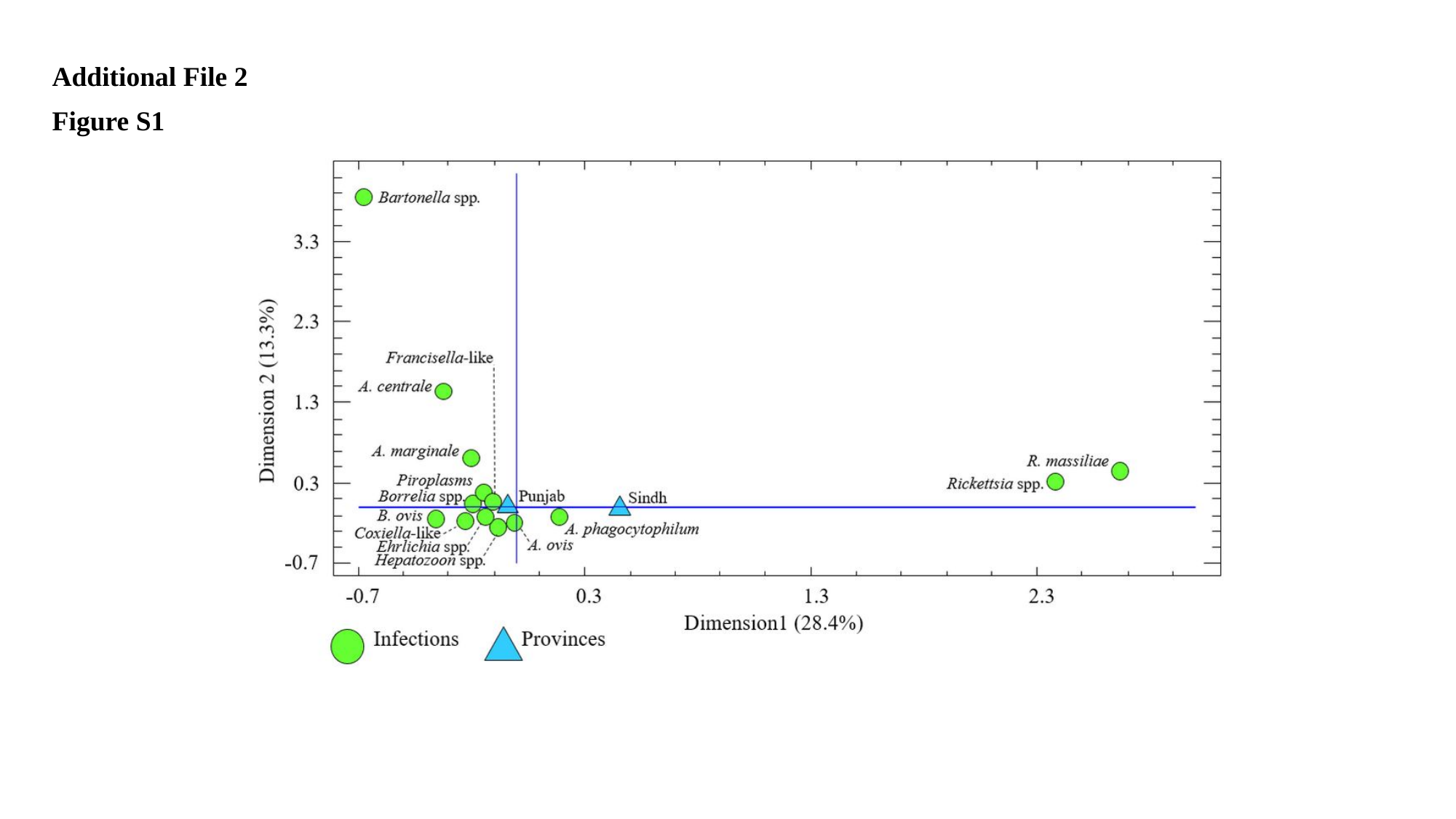

Additional File 2
Figure S1

Supplement: Supplementary file 2 — Additional file 2: Figure S1. Multiple correspondence analyses maps from the projections of the first two dimensions, showing the associations between infections (co-occurrence), and their distributions among six districts in Punjab and Sindh provinces of Pakistan. Percentage in each dimension (axis) indicates the fraction of the inertia that each principal component explains. Analyses are shown for correlation between infections and their prevalence in different provinces. [file 13071_2019_3862_MOESM2_ESM.pptx]

## Slide 1
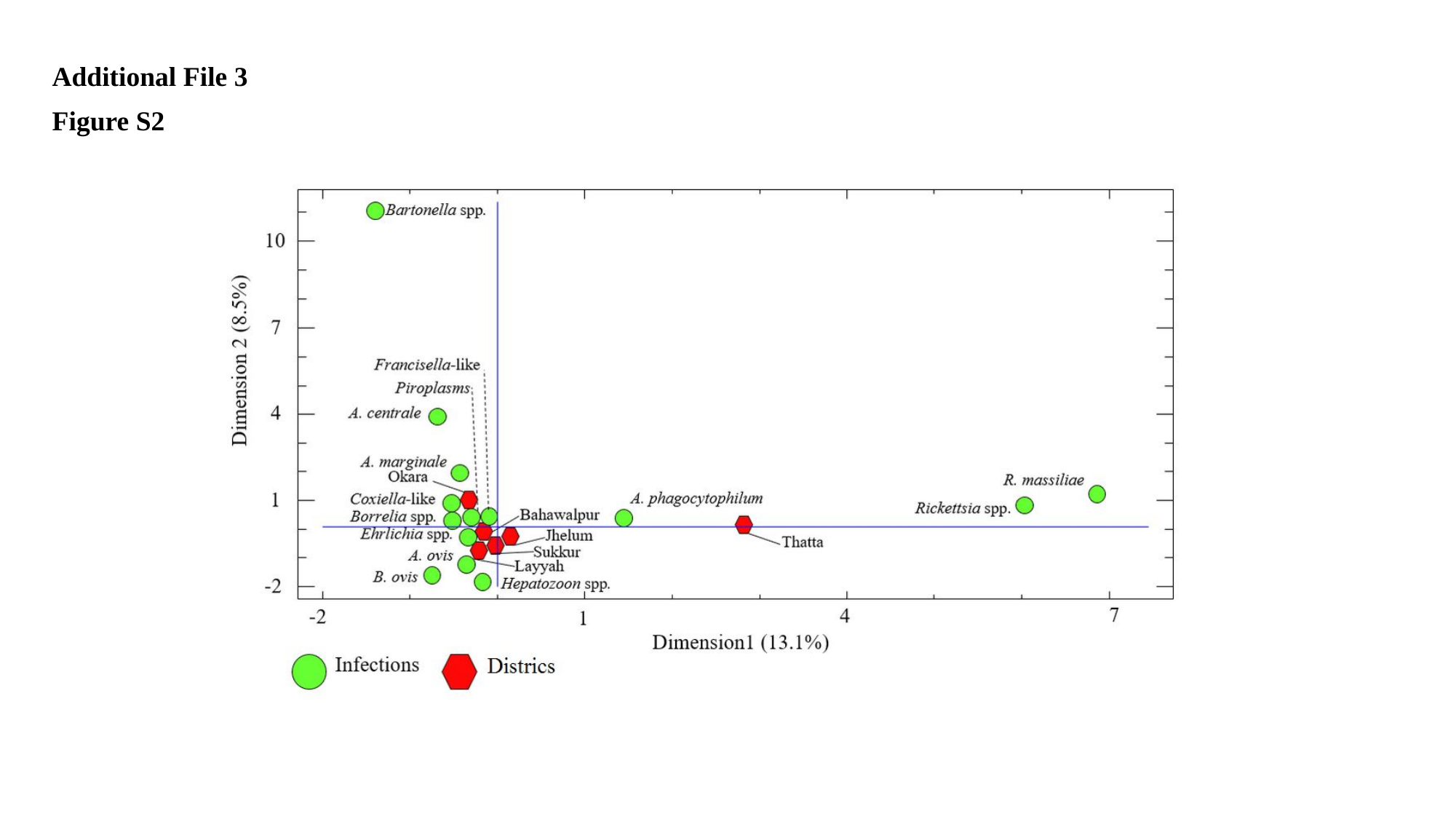

Additional File 3
Figure S2

Supplement: Supplementary file 3 — Additional file 3: Figure S2. Multiple correspondence analyses maps from the projections of the first two dimensions, showing the associations between infections (co-occurrence), and their distributions among six districts in Punjab and Sindh provinces of Pakistan. Percentage in each dimension (axis) indicates the fraction of the inertia that each principal component explains. Analyses are shown for correlation between infections and their prevalence in different districts. [file 13071_2019_3862_MOESM3_ESM.pptx]

## Slide 1
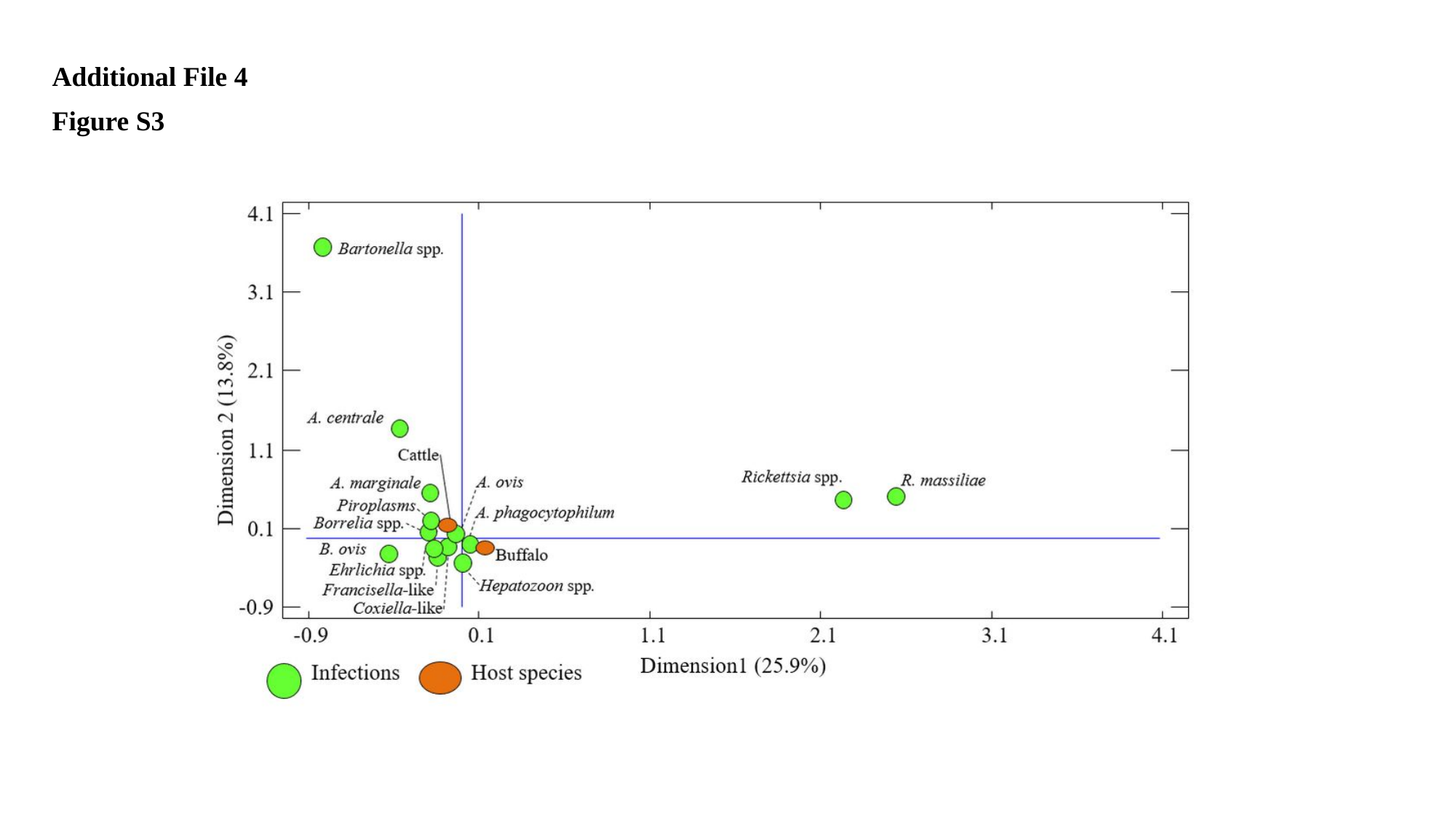

Additional File 4
Figure S3

Supplement: Supplementary file 4 — Additional file 4: Figure S3. Multiple correspondence analyses maps from the projections of the first two dimensions, showing the associations between infections (co-occurrence), and their distributions among six districts in Punjab and Sindh provinces of Pakistan. Percentage in each dimension (axis) indicates the fraction of the inertia that each principal component explains. Analyses are shown for correlation between infections and their prevalence in bovine host species. [file 13071_2019_3862_MOESM4_ESM.pptx]
